# The Investigation of Novel Dynamic Packing Technology for Injection Molded Part Quality Control and Its Production Stability by Using Real-Time PVT Control Method

**DOI:** 10.3390/polym14132720

**Published:** 2022-07-02

**Authors:** Yung-Hsiang Chang, Shia-Chung Chen, Yu-Hung Ting, Ching-Te Feng, Chi-Chuan Hsu

**Affiliations:** 1 R&D Center for Smart Manufacturing, Chung Yuan Christian University, Taoyuan City 320314, Taiwan; yunghsiangchang@gmail.com (Y.-H.C.); shiachun@cycu.edu.tw (S.-C.C.); eric.apple101@gmail.com (C.-T.F.); 2 Department of Mechanical Engineering, Chung Yuan Christian University, Taoyuan City 320314, Taiwan; g10102306@cycu.edu.tw; 3 Moldintel Co., Ltd., Taipei City 11070, Taiwan

**Keywords:** dynamic packing technology, injection molding, quality control

## Abstract

Injection molding is an effective mass production process for plastic, partly due to a number of advantages such as complex shape moldability, material selectivity, and a rapid process cycle. However, highly labor-based conventional production restrains the development of the industry. Experience-driven molding setups are used to trial the mold process, and also for quality checking the molded part for mass production. There is no effective solution for maintaining the production stability and defect-free adjustment. This study aimed to establish scientific packing pressure setup technology to optimize the molded part quality and the stability of consecutive production. The dynamic packing pressure setup technology for molded part quality and the process stability were investigated. This not only achieves the optimization of the packing pressure setup, but the stabilization of quality in mass production. Four major qualities were discussed in this study including tensile strength, regional deviation on shrinkage, total shrinkage, and warpage. The qualities improved by up to 3.9%, 92.9%, 41.9%, and 9.2%, respectively. A series of pilot runs of 300 cycles for two packing pressure control methods were tested to investigate the stability of the qualities. Dynamic packing pressure control improved the weight replication by 54%, reduced total shrinkage by 23%, and improved the warpage by 12%.

## 1. Introduction

Injection molding is used as an effective means of processing plastic products for mass production, and it possesses a number of process advantages such as diverse utilization of material, complex product shape moldability, and rapid mass production. However, numerous factors may cause product defects including warpage and uneven shrinkage due to inadequate processing conditions or material properties [1]. Product defects may only be found once the product has been produced or after a series of molding processes. In fact, all of these defects are hard to determine at the beginning of the process or during mass production, especially given that the parameters are set based on experience. The development of a scientific molding technology for online data-driven defect determination, the standardization and optimization of process conditions, and quality stabilization is essential.

**Figure 1:**
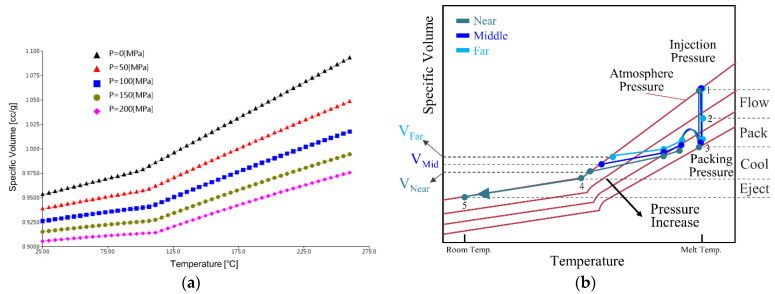
(**a**) PVT graphs for ABS PA756 and (**b**) PVT processing path for the entire injection molding process.

The pressure–volume–temperature (PVT) curve is derived from the pressure and temperature, and representing the fundamental material properties of polymers [2]. It can be measured by using three major test methods including the piston-die technique, confining-fluid technique [3], and online machine measurement [4,5]. Several researchers have improved the precision of the PVT diagram because the curve not only describes the entire molding process, but also depicts the quality of the molded part [6,7]. Figure 1a presents PVT curves (ABS PA756, ChiMei Corp., Tainan City, Taiwan) measured by a conventional polymer PVT tester based on the polymer heat expansion under different temperatures and pressures. Figure 1b presents the changes in the processing path of the PVT at each injection molding stage. The PVT property shows its potential in the development of a model that characterizes the molding process and the molded part quality. 

The PVT processing path control displays its potential not only for the molding parameter setup, but also for molded part quality control. The plastic components have defects for numerous complicated reasons; however, these are primarily generated by variations in shrinkage during the injection molding of plastic products. Studies show that many conditions affect the quality of the final product such as the molding parameters, including melt temperature, coolant temperature, cooling time [8], injection speed, and packing pressure. Chang et al. [9,10] revealed that the most influential parameters with regard to product quality are in the holding stage, packing pressure and packing time. You et al. [11] present a scientific method for molding parameter evaluation. The method covers most of the injection molding parameters. However, unstable real cavity temperature and pressure in consecutive mass production lead to increased fluctuations in product quality, as well as changes in product weight and cooling time [12]. Real data retrieval for molding stability and quality repeatability is crucial.

Sensor application is one of the solutions for real-time data retrieval in the process. The cavity sensor for measuring pressure and temperature is well utilized. Research on the influence of processing pressure and cavity temperature on the condition setup and part quality, including weight, dimensional stability, and molding defects has been conducted. Huang et al. showed that the V/P switch point has a significant influence on the part quality, which is point 2 on the PVT processing path [13]. Many V/P switch point control methods have been presented [14,15,16]. In fact, product weight, the stability of product dimensions, the mechanical properties, and surface quality are determined with the change in pressure variation [13]. Zhou et al. [17] established a product weight prediction model through a sensor embedded in the cavity for pressure data acquisition and achieved 0.015 g precision on product weight prediction. Moreover, a dynamic control method was proposed to reduce the weight deviation from 0.16% to 0.02% in only 5 cycles. This shows the potential if a dynamic control method was developed for part quality control. Gao et al. also showed better production quality control by replicating molding pressure [18]. Peak cavity pressure and integrated pressure were used for the quality control and product weight prediction [19,20]. On the other hand, a pressure sensor can not only be inserted into the cavity, but it can also be embedded in a delivery system such as a runner or gate. Tsai [21] presents the relationship between a runner and cavity pressure based on a sensor embedded in the runner to monitor its part quality. The studies described above reveal the possibility of collecting cavity pressure and temperature at the same time, and demonstrate its potential for product quality control.

In fact, the coupling data of the specific volume created by cavity pressure and temperature show its great controllability of the molded part quality and the stability of the mass production. Wang et al. demonstrated a method of product weight control using a thermal couple probe inserted at the same time as cavity pressure sensor. It had a great weight repeatability ranging from 0.05494 to 0.19683. The study shows that the product weight and other qualities are directly related to the P–T index [22,23]. Sheth and Nunn improved the dimensional stability by adjusting the setup pressure based on the melted temperature deviation [24,25]. These coupling data can be observed from the PVT processing path and the same idea also appliesd to specific volume control. As a result, if we can collect the real pressure and temperature data from the melted ones, the whole story of the practical PVT processing path can be described and the quality can be improved by specific volume control. Therefore, this study focuses on empirical data for the pressure and temperature to determine the PVT curve and establish the dynamic packing technology for achieving mass production stability control.

## 2. Materials and Methods

### 2.1. Sensor Position and Sample Preparation

**Figure 2:**
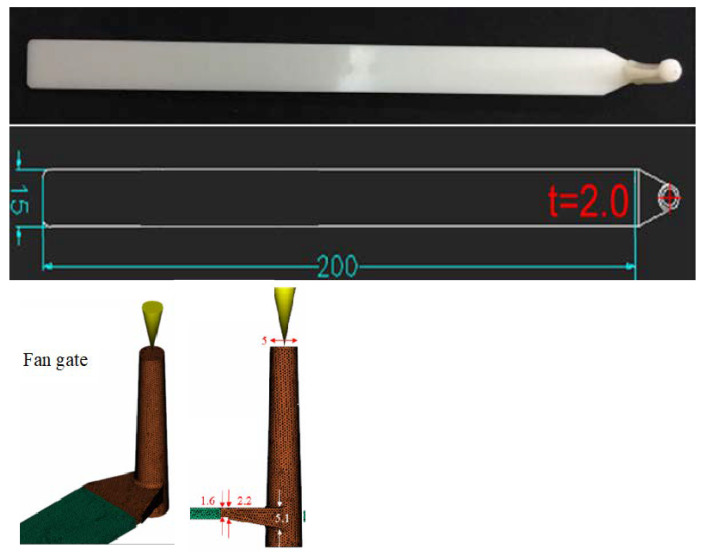
The strip model dimensions with a fan gate design.

**Figure 3:**
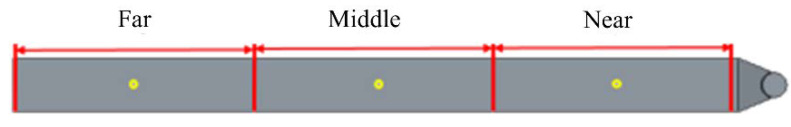
Sensor location for three sections of the model.

Three pressure and infrared temperature sensors were embedded in the mold. A set of sensors inserted at the same position in opposite mold surfaces were set up. This study is based on a strip model with a ratio of 100 for the flow-length to 2 mm thickness, as shown in Figure 2. The model is further divided into three sections with each set of sensors located in the center, which are labeled as near the gate (NG), middle, and far from the gate (FG). The mold design with the sensor fixer is shown in Figure 3. The purpose of the sensor fixer is to ensure the sensor is aligned with the mold surface.

### 2.2. Material and Machine

The injection machine, HSP 100 EH2 of Sodick Co., Ltd. (Yokohama, Japan) was employed in this study, based on the patented V-line structure of the injection unit for high precision on the injection volume and constant plasticized quality. ABS PA756 from Chi-Mei Chemical was used as the molding material. It is a commonly used engineering plastic in the plastic industry. The material was subject to 80 °C for 4 consecutive drying sessions in an HS-025LD drier supported by YePing Co., Ltd.

### 2.3. Data Collection and PVT Theory

The practical PVT curve was mapped using empirical data for the melt pressure and temperature in the mold cavity. The pressure data was obtained by a PRIAMUS eDAQ system and the temperature data was collected by a FUTABA^®^ EPT-001S. The sensor of the EPT-001S uses an optical fiber infrared method that can measure the actual melt temperature and it achieves high responsiveness to keep pace with the constantly changing melt temperatures. Both the pressure and temperature data were acquired by the molding data box of the StabMolding edge computing software powered by Moldintel Co., Ltd. StabMolding software has three main features, which are (1) data virtual inspection, (2) parameter optimization based on the practical setting condition, and (3) molded part quality control by updating the real-time parameters to the injection machine.

The PVT state transition from the Tait formula (Equations (1)–(9)) for temperature and specific volume was employed as the basic monitoring theory for StabMolding. It can be divided into two conversion stages, as shown below.

(1) $$v\left(T,p\right)=v_{0}\left(T\right)\left[1-Cln\left(1+\frac{p}{B\left(T\right)}\right)\right]+v_{t}\left(T,p\right)$$

(2) $$\bar{T}\equiv T-b_{5}$$

(3) $$T_{t}\left(p\right)=b_{5}+b_{6}p$$

where *v*(*T*, *p*) is real specific volume, $v_{0}$ is the specific volume at zero pressure, *C* is a constant (0.0894), $b_{5}$ is the glass transition temperature, and $b_{6}$ is the relative pressure of the glass transition temperature when *T* > *b*5

(4) $$v_{0}\left(T\right)=b_{1m}+b_{2m}\bar{T}$$

(5) $$B\left(T\right)=b_{3m}e^{-b_{4m}\bar{T}}$$

(6) v(T,p)=0

when *T* < *b*5

(7) $$v_{0}\left(T\right)=b_{1s}+b_{2s}\bar{T}$$

(8) $$B\left(T\right)=b_{3s}e^{-b_{4s}\bar{T}}$$

(9) $$v\left(T,p\right)=b_{7}e^{\left(b_{8}\bar{T}-b_{9}p\right)}$$

where $b_{1m}$–$b_{4m}$ represent the parameters of the melt-state material properties, and $b_{1s}$–$b_{4s}$ represent the parameters of the solid material properties. The formula demonstrates the real-time PVT processing path by converging online cavity pressure and temperature data collection. On the other hand, the real-time parameter updating to the injection molding machine is based on the standard OPCUA (Open Platform Communications Unified Architecture, an architecture that is widely used for data exchange from sensors to cloud applications) communicating protocol for every single cycle.

### 2.4. Design of Experiment

Since significance testing for all factors has already been discussed in a previous study [24] and the influence of packing had been confirmed, statistical verification was skipped in this article, and we focused on the PVT control method instead. The design of the experiment in this study is focused on a dynamic packing parameter setup based on a set of boundary conditions of molding parameters including melted temperature, mold temperature, injection speed, injection pressure, cooling time, and stroke. These parameters come from the professional molding condition guidance system made by Moldintel, Co., Ltd. (Taipei City, Taiwan), which is a theoretical plus practical experience-based calculation service. Four designs for packing setups are listed in Table 1.

The melted and mold temperatures in this study were in the middle temperature range recommended by the material supplier, and the injection pressure and speed were obtained by the optimum filling time derived from the experiment [10]. The part volume and thickness were used for injection stroke and the cooling time evaluation, which was calculated by Equation (10) [26].

(10) $$T_{c}=\frac{H^{2}}{\pi^{2}\alpha_{p}}\ln(\left(\frac{8}{\pi^{2}}\right)\times \left(\frac{T_{m}-T_{w}}{T_{e}-T_{w}}\right))$$

where $T_{c}$ is the cooling time, *H* is the part thickness, π is the circular ratio, $\alpha_{p}$ is thermal diffusivity, $T_{m}$ is melted temperature, $T_{e}$ is ejection temperature, and $T_{w}$ is the mold temperature.

### 2.5. Quality Measurement

**Figure 4:**
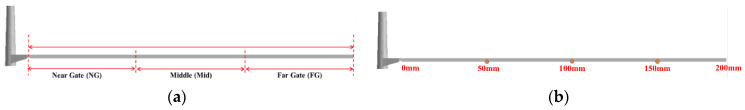
Measurement position for (**a**) shrinkage and (**b**) warpage.

Four major qualities are discussed in this study, including tensile strength, regional deviation on shrinkage, total shrinkage, and warpage. The measurement of shrinkage was calculated as the ratio of the original dimensions to the actual size in three sections as shown in Figure 4a. Warpage measurement was fixed at both ends of the product, before measuring the degree of product deformation, as shown in Figure 4b. The tensile strength was tested following the ASTM D638 datasheet for specimen preparation and experiment. Furthermore, the standard deviation (SD) was used to describe the specific volume deviation between the three sections, as shown in Equation (11).

(11) $$S=\sqrt{\frac{\sum^{\text{​}}\left(x_{i}-\bar{x}\right)}{n-1}}$$

where S is the standard deviation, $x_{i}$ is the specific sample value, $\bar{x}\;$is the sample average, and n is the number of samples.

## 3. Results and Discussion

### 3.1. Establishment of Practical PVT Monitoring

#### 3.1.1. Practical PVT Control Method Establishment

**Figure 5:**
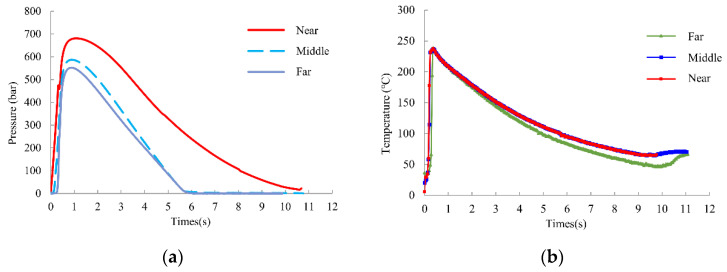
Three sections of (**a**) practical processing pressure curves and (**b**) real melted temperature curves.

PVT control technology is generally established using in-mold pressure sensors and infrared temperature sensors for pressure and melted temperature data acquisition. The practical processing pressure and melted temperature curve for the three sections are shown in Figure 5a,b. Both the pressure and temperature data were mapped by 25 Hz. The specific volume was obtained later by calculation of the Tait formula, and then drawn on Moldintel StabMolding software for the product quality exploration.

**Figure 6:**
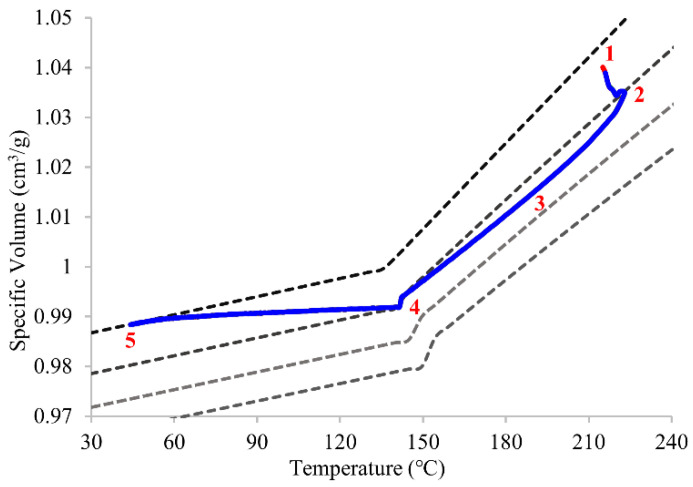
The practical PVT processing path for the whole injection molding process is depicted.

Figure 6 shows that the practical processing path displays the whole injection molding cycle including the injection stage (point 1 to 2), packing and holding stage (point 2 to 4), and cooling stage (point 4 to 5). Point 5 is also the ejection point, which represents the end of data acquisition because the product is ejected.

The practical processing path is mostly the same as the theoretical PVT path, with only slight differences at the filling stage. The pressure and temperature in the filling stage from point 1 to 2 are changed simultaneously, resulting in the discrepancy between the practical and theoretical PVT process. This can be observed from the temperature curve in Figure 5b. The temperature first increases around 10–15 °C by shear heating while the melted resin is injected into the cavity, and keeps the same cooling rate until it reaches the ejection temperature. Point 2 to 4 represents the pressure-driven processing path, which affects the final specific volume value, which is the most essential stage in this study for controlling product qualities. The point where the polymer solidifies is crucial to this study because that is the phase in which the product quality can be improved. For amorphous polymer, the glass-transition temperature is close to the inflection point between two slopes [27], that is, the polymer becomes solidified. In other words, point 4 results in the final specific volume of point 5. Once the processing path curve reaches 0 bar (the top of the PVT curve), it means that the polymer is frozen, and the polymer density in the cavity is determined.

#### 3.1.2. Packing Time Determination by PVT Processing Path

**Figure 7:**
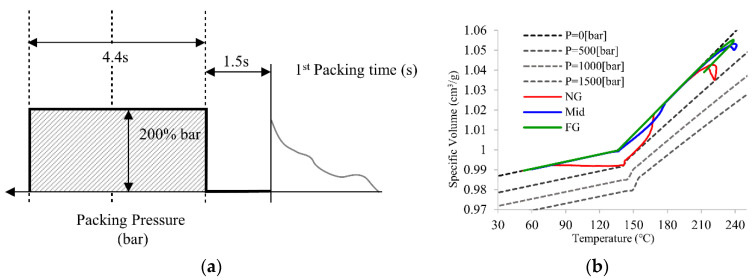
(**a**) Principle of intermittent packing pressure setup and (**b**) PVT processing path of principle of intermittent packing pressure setup for FG section.

The practical PVT processing path was used to determine the packing time. In order to optimize the packing time along the three sections, an intermittent packing pressure setting was used. After the filling stage is finished, the packing pressure ceases initially for 1.5 s, and resets the pressure to 200% of the cavity pressure of the end of the filling, see Figure 7a. Figure 7b shows the practical PVT processing path for three sections based on the intermittent pressure setup. The processing path at the FG section remains at 0 bar and after 1.5 s, the packing pressure ceased, which means the section is frozen and no longer affected by pressure delivery. The Mid and NG sections, however, are still affected by the reset packing pressure. The processing path increases after the pressure reset, which means these two sections remained unfrozen and can be controllable. As a result, 1.5 s is determined as the first packing time to FG section, and the concept of intermittent pressure setup can be used for the rest of the packing time determination.

**Figure 8:**
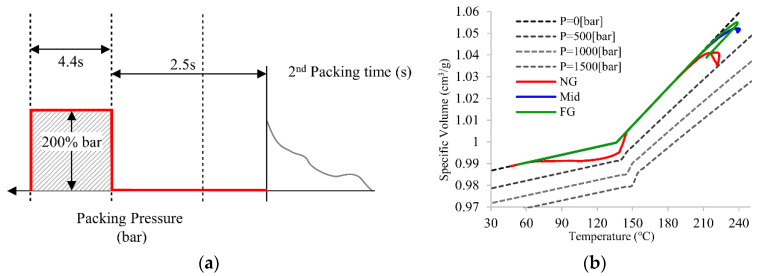
(**a**) Principle of intermittent packing pressure setup and (**b**) PVT processing path of principle of intermittent packing pressure setup for the Mid section.

Figure 8 presents the intermittent pressure setup concept and the PVT processing path for the Mid section. A total of 2.5 s is used for the intermittent packing pressure control, and the pressure is reset to 200% of cavity pressure at the end of the filling stage. Figure 8b shows that only the processing path of NG increases after the reset pressure control, which means these two areas of the part are frozen and are uncontrollable by pressure delivery. Therefore, the packing time for the Mid section is determined by 1 s, except the 1.5 s for the FG section. If we need to control the final specific volume (point 5) of the Mid section, the operative packing pressure is within this 1 s for the second packing time. Area NG, on the other hand, increases to around 145 °C, which illustrates that the area is unfrozen and controllable by pressure delivery.

**Figure 9:**
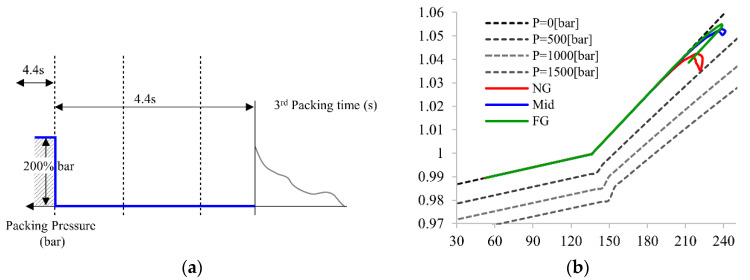
(**a**) Principle of intermittent packing pressure setup and (**b**) PVT processing path of principle of intermittent packing pressure setup for the FG section.

Figure 9 presents the intermittent pressure setup concept and its PVT processing path for the NG section. A total of 4.4 s is used for the intermittent packing pressure control, and the pressure is reset to 200% of the cavity pressure at the end of the filling stage. Figure 8b shows that all sections remain at 0 bar without influence after the packing pressure is reset. This illustrates that all areas of the part are frozen and are uncontrollable by pressure delivery. Therefore, the packing time for the FG section is determined as 1.9 s, except the 2.5 s for the FG and Mid sections.

#### 3.1.3. Packing Pressure Determination by PVT Processing Path to the Product Qualities

Traditionally, the packing pressure setup relies on the operator’s experience for pressure adjustment. However, polymer density along with the product is always uncertain, which results in changes in quality. In order to understand the influence of different stages of packing pressure, intermittent packing pressure was used to investigate the final specific volume and shrinkage. The design of the intermittent packing pressure experiment is shown in Table 2.

**Figure 10:**
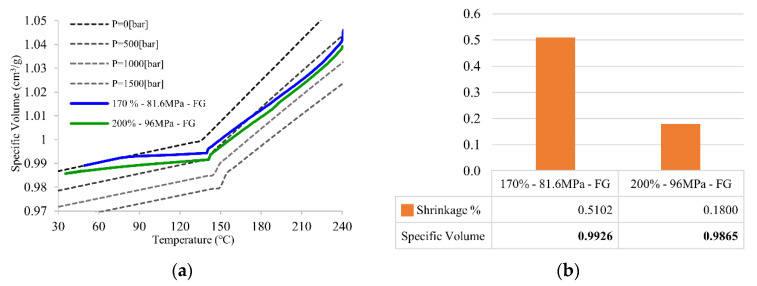
(**a**) PVT processing path of principle of intermittent packing pressure setup and (**b**) the final specific volume and its shrinkage for FG section.

Figure 10 shows the PVT processing path and the final specific volume with shrinkage in Group A of Table 2. The packing pressure setups in Group A focus on the effect of the first packing pressure along with the product with the pressure set right after the filling stage is finished. FG is the main section affected by the first packing pressure setup because the whole product remains unfrozen. The final specific volume and part shrinkage are directly related to the packing pressure. The specific volume decreases when the packing pressure increases, and the change in the specific volume results in shrinkage variation. This is because the deviation of the specific volume is equal to part shrinkage. Figure 10b shows that the higher packing pressure results in lower specific volume and shrinkage, which explains why the holding stage (point 2 to 4) is the most important stage in the PVT processing path [7].

**Figure 11:**
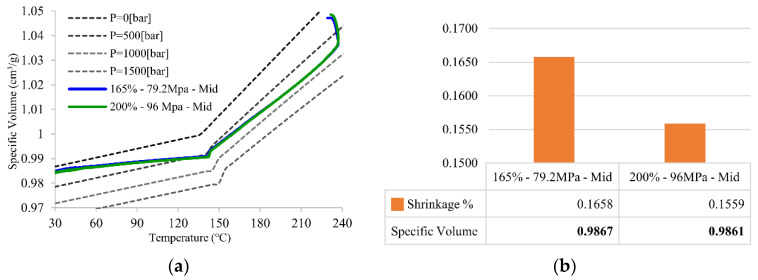
(**a**) PVT processing path of principle of intermittente packing pressure setup and (**b**) the final specific volume and its shrinkage for Mid section.

Figure 11 shows the PVT processing path and final specific volume with shrinkage in Group B of Table 2. This design set focuses on the effect of second packing pressure along with the product with the pressure set right after the first packing pressure is finished. The Mid section is the main section affected by the second packing pressure setup because the Mid and NG areas remain unfrozen. Figure 11b shows the same trend as Group A, where the specific volume decreases with the increase in packing pressure. The specific volume in the Mid section presents a similar result as well as the PVT processing path. The path’s progress is similar because the polymer density along the part must be homogeneous; even with high density in the Mid section at first, the polymer will push the polymer toward the low-density area or to the area that remains unfrozen, such as the NG sections.

**Figure 12:**
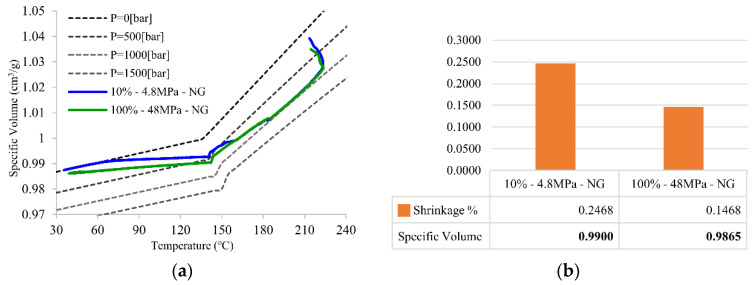
(**a**) PVT processing path of principle of intermittent packing pressure setup and (**b**) the final specific volume and its shrinkage for NG section.

Figure 12 shows the PVT processing path and final specific volume with shrinkage in Group C of Table 2. The packing pressure setups in this group focus on the effect of the third packing pressure along with the product with the pressure set right after the second packing pressure is finished. NG is the main section affected by the last packing pressure setup because the whole product is fully solidified. Figure 10b shows the same tendency as the two packing pressure settings before; higher packing pressure brings about lower specific volume and shrinkage.

Figure 10, Figure 11 and Figure 12 illustrate the same tendency in regard to the practical PVT processing path, final specific volume, and shrinkage. Larger packing pressure results in the lower specific volume and shrinkage. On the other hand, the pressure is directly related to the processing path. In order to minimize product shrinkage, the path can be further controlled by the packing pressure to reduce the final specific volume. Intermittent packing pressure was used for the packing time setup and the influence of packing pressure on the different sections of product shrinkage. Shrinkage varies with the packing pressure change and the deviation in the final specific volume. Moreover, final specific volume can be an index for molded part quality estimation or parameter adjustment.

Section 3.1.2 and Section 3.1.3 confirm that the packing time determination and the packing pressure have an influence on the product. The earliest packing pressure affects the farthest section and the latest pressure setting affects the section nearest to the gate, which means FG is affected by the first packing pressure, the Mid section is affected by the second packing pressure, and NG is affected by the third packing pressure. Following the confirmation of the potential for packing pressure and time optimization, in Section 3.2, we propose a new dynamic packing technology to improve product qualities through practical PVT processing paths.

### 3.2. Dynamic Packing Technology Establishment

#### 3.2.1. Optimization of Packing Pressure and Packing Time for Dynamic Packing Technology

**Figure 13:**
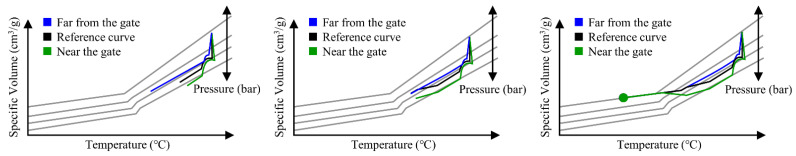
Optimization concept for packing pressure adjustment by the PVT processing path.

Due to the packing time determination process via the intermittent packing pressure setup, three packing times were confirmed for the development of the dynamic packing technology; these were 1.5 s for the first packing time, 1 s for the second packing time, and 1.9 s for the third packing time. A practical PVT processing path was used as the standard for packing pressure adjustment. The concept of dynamic packing technology aims to achieve a consistent final specific volume in different sections along the part. From the results above, the specific volume presents the polymer density within the cavity. Uniformity of polymer in the part results in better product shrinkage and product qualities. In order to obtain the final specific volume, three sections of the processing path control are used to adjust the packing pressure through the observation of the temporary specific volume as shown in Figure 13. The specific volume of each section is determined at the end of the packing time. Pressure delivery only affects the area that remains unfrozen.

Figure 13 shows the temporary specific volume of three sections at the end of the first packing time. After this packing time, the specific volume of FG is no longer changeable, that is, if we would like to fit all three specific volumes, the 2nd and 3rd packing pressures need to be adjusted to match together. The 2nd packing pressure reduces right after the end of the 1st packing time to fit the Mid section of the PVT processing path to the FG one. Following the same procedure, the three processing paths fit to the same final specific volume eventually.

#### 3.2.2. Investigation of Dynamic Packing Technology for Product Qualities

Four types of packing strategies for molded part quality and the practical PVT processing path were investigated for tensile strength, regional deviation on shrinkage, total shrinkage, and warpage. These four packing strategies derive from the most used packing setups =based on operator experience, that is, single packing (Single), sequential decrease in packing (Design 1), intermittent packing (Design 2), and the dynamic packing setup (Design 3).

Single packing is the easiest method for the injection molding process. However, most dimensions of the injection molded part are unsuitable for using single packing only, especially if the product needs a long holding stage for the thicker parts, single packing with high pressure may cause flash defects or overpacking. Therefore, the sequential packing process is utilized for the packing setup by the majority of operators. Design 2, the intermittent packing process is a special packing technique where the product requires a long packing pressure delivery and cannot sustain high pressure at the beginning. However, the experience-driven packing techniques above are difficult to realize along the part in a practical situation. In order to develop a scientific packing technique for uniform polymer density in the entire product, Design 3, the dynamic packing technique based on the concept described in Section 3.2.1 is proposed.

**Figure 14:**
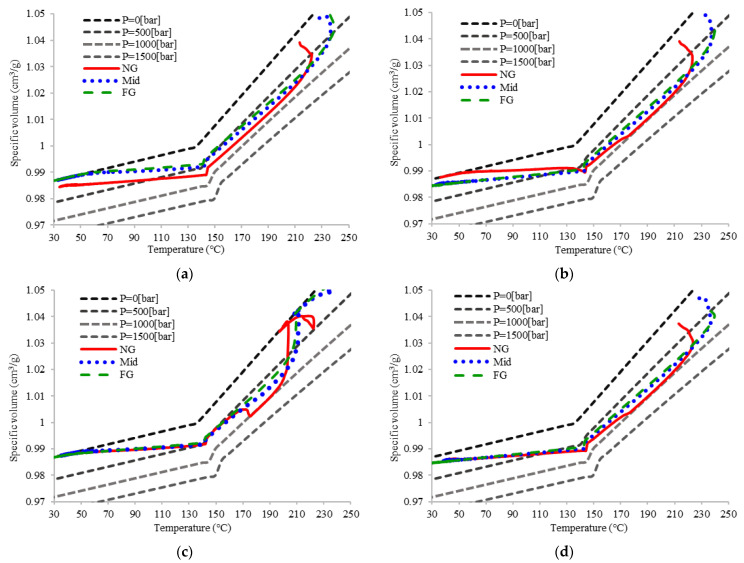
PVT processing paths for (**a**) single packing, (**b**) sequential packing setup, (**c**) intermittent packing setup, and (**d**) dynamic packing technique.

Figure 14 shows the PVT processing path for four types of packing strategy, and presents its relationship under different control methods. Each packing setup results in a different final specific volume for the three sections along the product. Moreover, the most effective period for packing pressure control is from point 2 to 4. After point 4, the molded part in the cavity cools naturally until fully solidified. Figure 14a displays the great discrepancy in the final specific volume for three sections influenced by the single packing pressure setup. The high pressure delivered from NG to FG causes the pressure difference along the product with polymer condensed near the gate. Consequently, the final specific volume for NG is much smaller than the specific volume at the Mid and FG area. The PVT path for Design 1 causes the same discrepancy in the regional specific volume. Traditional sequential decreasing packing setup may result in the smaller specific volume by reducing the last packing pressure setup. The product shrinkage is directly related to the final specific volume, the smaller the specific volume, the higher product shrinkage. This is the idea that supports the development of the dynamic packing technology. If the packing pressure adjusts based on the specific volume change, three sections of the polymer density can finally fit together, resulting in even product shrinkage.

Based on the idea of real-time processing path monitoring, Design 2 and 3 are the dynamic packing pressure controls. Figure 14c presents the PVT processing path of the intermittent packing pressure setup, which is used for the complex or high-flow length product dimension. The main reason for this setting strategy is that by using high pressure delivery to the end of the part, a thicker frozen layer is established. However, serious unbalanced pressure distribution may be induced. Dynamic packing technology by practical PVT processing path monitoring is first introduced. After the initial packing is paused, the pressure is soon reset. The first packing time for FG area is reduced because the packing ceased for a few moments. Higher specific volume for all three sections is observed compared to Design 3. This is because a little less packing pressure can affect the product after the formation of a thicker frozen layer. Although the specific volume is higher, in the end, the three sections are end matched by the pressure adjustment based on processing path control. In order to further suppress the polymer density, Design 3 is proposed to optimize the final specific volume of the part. The results show that the specific volume of Design 2 is smaller than the one of Design 3. This is crucial because product quality is directly related to the final specific volume. The four qualities are further discussed.

**Figure 15:**
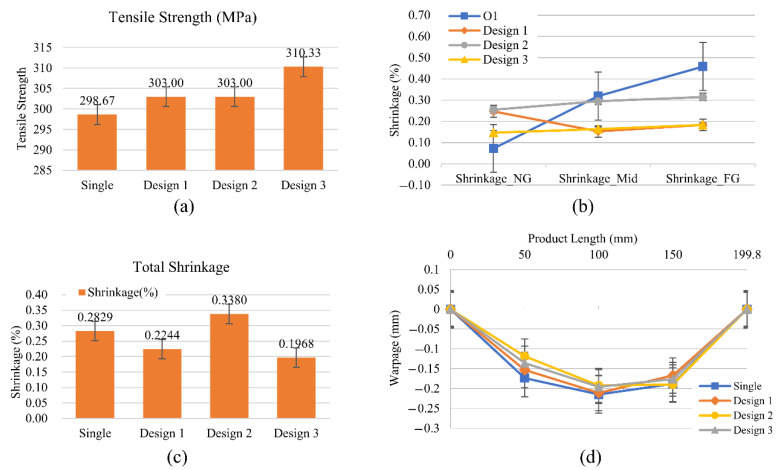
(**a**) Tensile strength, (**b**) regional deviation of shrinkage, (**c**) total shrinkage, and (**d**) warpage.

Figure 15 presents the four product qualities related to the packing designs. It shows that the smaller specific volume results in higher tensile strength and less total shrinkage. The tensile strength increases because the smaller specific volume indicates that more polymer molecules are condensed within the product and this compact formation also reduces the total shrinkage. On the other hand, polymer density discrepancy is one of the reasons for warpage formation. The more uniform specific volume for the three sections results in less shrinkage discrepancy and warpage. At the same time, dynamic packing technology for Design 2 and 3 present the potential for better product qualities in all four indexes. The tensile strength, regional deviation on shrinkage, total shrinkage, and warpage for Design 3 compared to the single packing setup were improved by up to 3.9%, 92.9%, 41.9%, and 9.2%, respectively.

### 3.3. Quality Repeatibilty on Consecutive Molding Process by Dynamic Packing Technology

P-t control and dynamic packing technology compared to the uncontrolled consecutive molding process were investigated. Natural mold temperature change was used to reproduce the real molding process in which the mold temperature changes over time with melted resin injected into the cavity.

**Figure 16:**
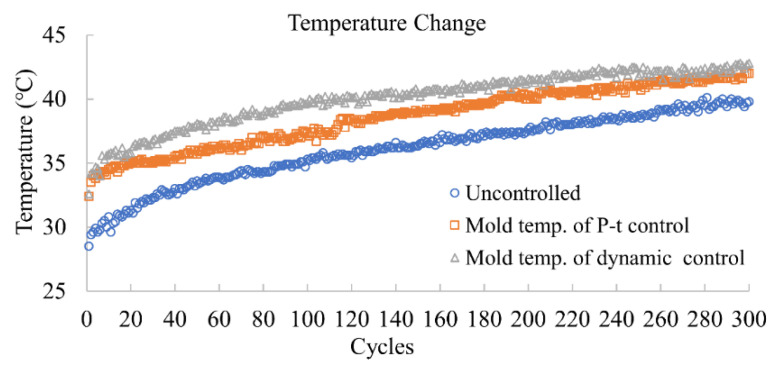
Natural mold temperature changes over 300 cycles of consecutive molding.

Figure 16 shows the temperature change in 300 cycles for three different quality controls. P-t control is the most widely used method for product weight control [17,18,20,21].

**Figure 17:**
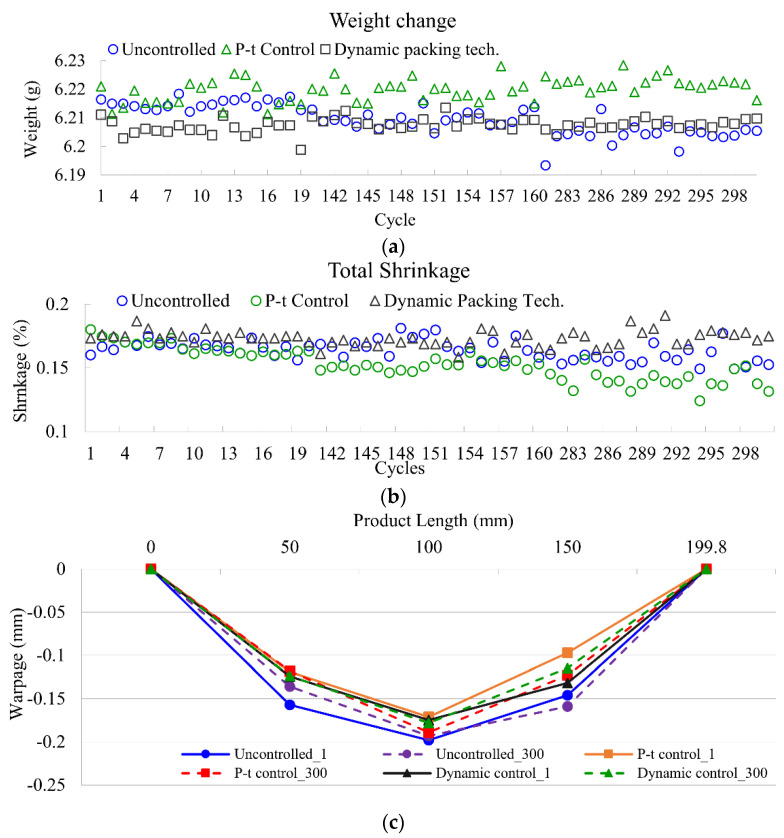
The results for (**a**) product weight, (**b**) total shrinkage, and (**c**) warpage for three control methods.

Figure 17 shows the results for weight, total shrinkage, and warpage using three different quality controls. Figure 17a shows that the product weight for the uncontrolled group decreased by 0.03% from cycle 1 to 300. This is mainly because the heat expansion of polymer molecules injected into the cavity. In addition, a lower pressure integral for the uncontrolled group was observed, which was verified by the weight result. P-t and dynamic packing technology, on the other hand, remain steady for the product weight. The consistent product weight of dynamic packing technology resulted from the automatic packing pressure adjustment using the concept outlined in Section 3.2.1.

Total shrinkage, however, shows a different story for P-t control. Although Pt control presents outstanding weight control, the compensation of the packing pressure in only one section produces a high polymer density area. This can further result in the uneven specific volume along the part, as a result, the warpage is increased. The total shrinkage for the P-t control from cycle 1 to 300 decreases by 0.03%, and the warpage is increased by 11%. The total shrinkage and product warpage for the uncontrolled group is relatively steady compared to P-t control, which presents the largest warpage for all three groups.

Dynamic packing technology displays stability on total shrinkage. This is because the packing pressure for the three sections is adjusted dynamically through practical PVT processing path monitoring. The consistency of the final specific volume in every cycle results in uniform regional shrinkage as well as the product warpage. The total shrinkage and warpage were improved by 23% and 12%, respectively, compared to the uncontrolled group.

## 4. Conclusions

A practical PVT control method is established as a reference for scientific parameter setting process and quality optimization. The real cavity pressure and melted temperature are obtained for a further PVT processing path calculation. The intermittent packing pressure setup is an effective method for packing time determination by using the practical PVT processing path change. On the other hand, the scientific packing pressure was also investigated. The 1st section of packing pressure affects the farthest area of the specimen, until the 3rd section of packing pressure for the nearest area close to the gate. Product quality varies with the packing pressure and the final specific volume change. Moreover, it shows the influence of final specific volume change on product quality.

Four major product qualities were investigated along with the practical PVT processing path and the specific volume change. Packing pressure setup is directly related to the practical PVT processing path, and has a great influence on the final specific volume of the product. Dynamic packing technology was determined based on four packing pressure setups. Moreover, the constant specific volume from the dynamic packing technology for three sections led to the best product quality in regard to all properties. The tensile strength, shrinkage deviation of three sections, total shrinkage, and warpage were improved by 3.9%, 92.9%, 41.9%, and 9.2%, respectively.

Three different control methods were determined for the consecutive quality change in 300 pieces in a pilot run molding process. Dynamic packing technology resulted in quality repeatability in three quality indexes, product weight, total shrinkage, and warpage. Although the product weight derived from P-t control is constant, it can induce uneven pressure distribution along the flow path to the product, and also cause unbalanced specific volume. Consequently, product weight maintained great consistency except for total shrinkage and warpage. The product weight, total shrinkage, and warpage compared to traditional non-controlled processes were improved by about 54%, 23%, and 12%, respectively.

## Figures and Tables

**Table 1:** Experimental parameters.

	Single Packing	Design 1	Design 2	Design 3
Melted temp. (°C)	220			
Mold temp. (°C)	30			
Filling time (s)	0.7			
Cooling time (s)	5.4			
1st Packing pressure	170%	200%	1%	200%
1st Packing time	4.4	1.5	0.5	1.5
2nd Packing pressure	null	130%	220%	Dynamic
2nd Packing time		1	1	1
3rd Packing pressure		90%	Dynamic	Dynamic
3rd Packing time		1.9	1	1.9
4th Packing pressure		null	Dynamic	null
4th Packing time			1.9	

**Table 2:** Experimental parameters for packing pressure determination.

Mold Temperature	30 °C					
Filling time	0.7 s					
Cooling time	5.4 s					
Group	A1	A2	B1	B2	C1	C2
1st Packing pressure	170%	200%	200%			
1st Packing time	1.5s					
2nd Packing pressure	null		165%	200%	165%	
2nd Packing time			1s			
3rd Packing pressure			null		10%	100%
3rd Packing time					1.9s	
